# Pathogenomics of Endometriosis Development

**DOI:** 10.3390/ijms19071852

**Published:** 2018-06-23

**Authors:** Vladislav Baranov, Olga Malysheva, Maria Yarmolinskaya

**Affiliations:** D.O.Ott Institute of Obstetrics, Gynecology and Reproductology, Saint-Petersburg 199034, Russia; omal99@mail.ru (O.M.); m.yarmolinskaya@gmail.com (M.Y.)

**Keywords:** endometriosis, developmental pathway, pathogenomics, mesenchymal stem cells

## Abstract

For over 100 years, endometriosis, as a chronic, estrogen-dependent, inflammatory, heritable disease affecting approximately 5–10% of women in reproductive age has been the focus of clinicians and scientists. In spite of numerous environmental, genetic, epigenetic, endocrine, and immunological studies, our knowledge of endometriosis is still fragmentary, and its precise pathophysiology and pathogenomics remain a mystery. The implementation of new technologies has provided tremendous progress in understanding the many intrinsic molecular mechanisms in the development of endometriosis, with progenitor and stem cells (SCs) of the eutopic endometrium as the starting players and endometriotic lesions as the final pathomorphological trait. Novel data on the molecular, genetic, and epigenetic mechanisms of the disease are briefly outlined. We hypothesize the existence of an endometriosis development genetic program (EMDP) that governs the origin of endometrium stem cells programmed for endometriosis (1), their transition (metaplasia) into mesenchymal SCs (2), and their invasion of the peritoneum and progression to endometriotic lesions (3). The pros and cons of the recent unifying theory of endometriosis are also discussed. Complex genomic and epigenetic interactions at different stages of the endometriosis process result in different forms of the disease, with specific features and clinical manifestations. The significance of the EMDP in elaborating a new strategy for endometriosis prediction, prevention, and treatment is discussed.

## 1. Introduction

Endometriosis is a common disorder affecting 5–10% of women of reproductive age. By clinical manifestation, it corresponds to chronic, estrogen-dependent inflammation mitigated by the growth of endometrium-like tissue in sites other than the uterine cavity, most commonly in the pelvic cavity. Although studied for a century, many aspects of the pathophysiology and developmental pathogenetics of the disease still remain obscure, and practical achievements in the prediction, prevention or treatment of endometriosis remain rather illusive to date [1,2]. A detailed understanding of the molecular mechanisms underlying endometriosis is also far from complete. Meanwhile, spectacular achievements in molecular diagnostics and system genetics in studies of this common disease have provided a huge bulk of useful information regarding the genetic aspects of endometriosis and the molecular mechanisms of its origin and development [3,4]. Many theories and attractive hypotheses on the pathogenesis of endometriosis are known but they are rather contradictory. Genetic, endocrine, environmental, immune, and epigenetic factors have been studied in numerous articles to explain the mechanistic basis of the origin and development of endometriotic lesions [5,6]. Conspicuous progress in this area has been achieved during the last decade, mainly due to the identification of new candidate genes and numerous SNPs (single nucleotide polymorphism) tightly associated with endometriosis [6], of genetic and epigenetic mechanisms of its regulation [5,7], and of endometrial stem cells [8], and to transcriptome and miRNA analyses of the endometrium and endometriotic cells [9,10]. The contribution of epigenetic and genetic factors in the pathogenesis of endometriosis has been described in many exhaustive reviews [3,4,10,11,12].

Studying endometriosis as a problem of developmental genetics is a principal goal of the present paper. The origin of endometriotic cells and the genetic and epigenetic factors contributing to the initiation and growth of endometriotic lesions are briefly reviewed. We hypothesize the existence of a special endometriosis development program (EMDP) which switches on in the progenitor SCs of the endometrium and in SCs descended from the Mullerian duct. EMDP suggests that the cells are prone to giving rise to endometriosis partly through endometrial–mesenchymal transition, their invasion into the peritoneum lining, and differentiation and growth into endometriotic lesions.

Classical embryology and developmental biology postulate that each morphogenetic event has its own critical and sensitive period (SP) which displays a heightened sensitivity to internal and external stimuli [13]. According to further molecular studies, the critical periods precede visible morphogenetic reactions and correspond to massive genome reprogramming [14]. The suggested SPs of EMDP should be considered a suitable timeframe for the prediction and treatment of endometriosis. The epigenetic landscape of endometriosis reflects the complex interactions of genetic and epigenetic factors, which underlies the pathogenomics of endometriosis [15], creates a unique EMDP, substantiates endometriosis clinical manifestations, and provides clues for a personalized treatment of this disease.

## 2. Key Stages of Endometriosis Development

### 2.1. Stem Cells in the Pathogenesis of Endometriosis

SCs are defined as undifferentiated cells which possess both self-renewal and differentiation abilities [16]. The possibility for extra-uterine SC to progress into endometriotic lesions may explain endometriosis developing in distant sites such as the lungs. They also support the theory suggesting that SC may travel via lymphovascular spaces [17]. Finding the stemness-related genes, such as *OCT4*, *SOX2*, *SOX15*, *NOTCH1*, *TWIST1*, and others, expressed in endometriotic lesions, may help show that the mechanisms determining the self-renewal rates and SC fates are deregulated in endometriosis, leading to altered SC behavior [18].

According to initial studies, the multi-site origin of endometriotic SCs was repeatedly suspected [3,6,19]. Different types of endometrial SCs were hypothesized, such as endometrial SCs in the peritoneum and pelvic cavity (1), resting embryonic cells descendent from the Mullerian duct (2), SCs in menstrual debris (3), coelomic epithelial cells after metaplasia (4), and mesenchymal bone marrow SC (bmSCs) in inflammation sites in the peritoneum (5). It was postulated that SCs that originated from bone marrow SCs could also be attracted in the human endometrium, but their participation in endometriosis should be proven [3,19]. Several different types of SCs have been suggested in the endometrium itself, including progenitor cells of the endometrium, mesenchymal stem cells, and endothelial stem cells [16,20]. Under appropriate conditions, SCs shed with menstrual blood can differentiate into typical mesenchymal lineages [21]. Thus, although the exact location of endometrial SCs still needs to be explored, some findings suggest that the inner basal layer resting on the myometrium at the endometrium–myometrium interface and known as the “junctional zone”, should be treated as a preferential site for the endometrial SC niche [16,22]. Also, bmSCs in the endometrium could contribute to all stem cell kinds in the endometrium [19,23] The existence of own SCs in the endometrium is also postulated, although the specific markers to identify endometrial SCs have not yet been established [19,24].

As might be inferred, little doubt is left with regard to the SC origin of endometriosis. Whether they SCs in the endometrium are endometrial by origin or come from other sources like the bone marrow, peritoneum, or some other tissues, remains unknown. Meanwhile, two major sources of endometriotic SCs should be considered: SCs disseminated throughout the peritoneum lining the pelvic cavity during embryogenesis of the female reproductive tract (endometriosis of extrauterine origin) (1), and SCs from the endometrial layer (endometriosis of intrauterine origin) (2). The hypothesis of the extrauterine origin of endometriosis from mesenchymal SCs disseminated during embryogenesis that infested the epithelium lining of the pelvic cavity has recently received major support in the novel “unifying theory” of endometriosis pathogenesis [24]. More details of this hypothesis will be given in the Discussion. The second hypothesis is in line with the well-known hypothesis by Sampson (1927), which postulates that the endometriosis originates from the menstrual cells of endometrial tissue disseminated in the pelvic cavity [25].

### 2.2. Initial Stages of Endometriosis

The most intriguing problem of endometriosis pathogenesis concerns the molecular mechanisms underlying the acquisition of tumor-like properties by otherwise normal SCs. According to the “uterine origin” and the “extrauterine origin” hypotheses, metaplasia of the endometrial (epithelial) cells into mesenchymal cells (so-called epithelial–mesenchymal transition—EMT) may play a key role in the pathogenesis of endometriosis [26].

EMT is a biologic process during which polarized epithelial cells by consecutive changes get a mesenchymal cells phenotype. EMT plays a role in a series of biological settings, such as implantation and embryogenesis and pathogenesis of malignant tumors, and is also associated with wound healing, tissue regeneration, and organ fibrosis [27]. The molecular mechanisms of EMT in epithelial cells involve the functional loss of E-cadherin, desmoplakin, and mucin-1 and increased expression of such mesenchymal markers as N-cadherin, smooth-muscle actin and ohers [28]. Cells of different origin can enter EMT leading to development of endometriosis. These cells can be peritoneum epithelium cells (as according to the metaplastic theory of development of endometriosis), endothelial cells, and also epithelial cells of the endometrium [26]. The molecular mechanisms of EMT have now been studied in detail [18].

Main inducers of EMT are well known [27]. Chronic injury and subsequent inflammation can trigger EMT through the release of some cytokines, such as TGF-β, PDGF, EGF, and FGF-2. A number of authors have reported that that the TGF-β level have increased in peritoneal fluid and serum of women with endometriosis compared to healthy women [29]. Other inducers of EMT are hypoxia and other factors (i.e., the Ras–MAPK (mitogen-activated protein kinase) pathway) leading to hyperexpression of hypoxia-induced factor-1 (HIF-1A) [26].

The principal role in the metaplasia of the endometrial epithelium might be attributed to the *TWIST1* gene (Twist family basic-loop-helix transcription factor 1). It was identified as a key regulator of mesoderm development and later have been implicated in many human diseases. The expression of *TWIST1* is closely related to tumor aggressiveness and metastatic potential [30]. Twist1 has also been shown to function as a key regulator of EMT. Driven by HIF-1, Twist1 realizes its developmental functions by governing cell movement and tissue reorganization [31]. The molecular mechanisms underlying EMT induced by TWIST in epithelial cells involve functional loss of E-cadherin (CDH1) in the eutopic endometrium of endometriosis patients. Reduced level of cadherins accompanied by excessive expression of metalloproteases (MMP) genes provide favorable conditions for cell migration. A mechanosensitive transduction pathway involving β-catenin specifies the early mesodermal conservation, which is required for Twist mechanical identity. Thus, transient hypoxia and mechanical tension switch on EMT through the activation of *TWIST1*. The expression of doublecortin- and Ca^2+^/calmodulin-dependent protein kinase-like protein-1 (DCAMKL-1), which is known to regulate *TWIST1*, *Myc*, *KRAS*, and other factors, was also recently discovered [18]. Furthermore, it has been pointed out that there might also be some imbalances in micro-RNAs (miRNA) in women with endometriosis, enhancing cell invasiveness due to impaired miR-145 or promoting proangiogenic factors due to the downregulation of miRNA-199a-5p or extracellular matrix regulator miRNA 29a, significant downregulation of mir-200b in the endometrium and in peritoneal lesions, and regulation of *HOX* genes family miRNA196 [10]. Over 600 different miRNAs associated with endometriosis at each stage of development are known so far. The available results in miRNA studies of endometriosis are rather contradictory and need thorough revision [10]. The significant heterogenicity of endometriotic lesion samples is considered a major problem when analyzing the miRNA signatures of whole endometriotic lesion biopsies [4,9,10].

Thus, during the dormant stage of endometriosis, there are some cells of endometrial origin which might potentially contribute to the growth of endometriotic lesions. The latter is regulated by the activation of specific transcription factors induced by transient hypoxia, chronic inflammation, and mechanical tension switch. The cells lose their polarity and contacts and acquire the migratory and invasive abilities of mesenchymal stem cells. The expression of the *MYC* and *CCND1* (cyclin D1) genes leads to high proliferative activity, while the upregulation of *BCL2* reduces apoptosis and prolongs survival. Thus, as a consequence of EMT, epithelial cells lose their specific features as well as their integrity and acquire mesenchymal traits linked to increased invasion and migration properties [18]. Under appropriate hormonal and immunological stimulation, the SCs shed into the peritoneal cavity during retrograde menstruation gain abilities for invasion, implantation, and growth [19]. It should be reminded that endometriosis might also stem from the stromal cells of the endometrium itself, although their capacity for proliferation, invasion, and endometriotic lesion growth are still not known. There are some data showing that SCs derived from the menstrual blood debris in an endometriosis patient also showed altered SC functions, which favor the establishment of endometriotic implants [16].

### 2.3. Invasion of Endometriotic SC

The basic signs of endometriosis development include endometriotic SC invasion in the peritoneum, and their proliferation and differentiation into endometriotic lesions. Women with endometriosis are known to have increased macrophage activity, decreased cellular immunity, and reduced natural killer cell counts [8]. Thus, following retrograde menstruation, the immunodeficient condition prevents the clearance of the menstrual debris from the peritoneum, making the ectopic endometrial cells persist [32]. The latter induce inflammation, recruit macrophages and leukocytes, and, thereby, promote the development of endometriosis [33].

The molecular profiling of the eutopic endometrium from endometriosis patients suggests functional alterations in the genes that facilitate proliferation, implantation, and survival of the endometrial tissue in the peritoneal cavity, thus supporting endometriosis pathogenesis from the altered eutopic endometrium. Inflammatory, immune, and angiogenic responses as well as apoptosis reactions are altered in the eutopic endometrium of affected women, thus favoring the survival and the maintenance of ethe ndometriotic tissue [34].

The relocation of SCs from the eutopic endometrium to ectopic sites in the pelvic cavity potentiates the release of several chemokines and cytokines which favor revascularization and thus allow the development of endometriotic lesions [17]. Comparisons between SCs in the eutopic endometrium and ectopic SCs in the peritoneal cavity by analyzing their phenotypes and gene expression of pro-inflammatory cytokines, migration markers, and angiogenic factors proved the increased levels of these molecules, accompanied by the reduced levels of anti-inflammatory cytokines such as TGFβ. The increased levels of pro-inflammatory cytokines such as interleukin-6 (IL-6) and interferon-γ (IFNγ) and the presence of the migration markers matrix metalloproteases (MMP)-2, -3, -9 and of the proangiogenic vascular endothelial growth factor (VEGFA) in ectopic tissue indicate that the abnormal behavior of ectopic mesenchymal SCs may suppress the immune system and enhance angiogenesis [35]. The increased expression of MMPs would also be useful for the ectopic endometrial tissue to activate invasion.

The processes of implantation of endometriotic SC onto the peritoneum and endometriotic lesion growth obviously require angiogenesis. Several studies have reported an increase in VEGFA level in the serum and peritoneal fluid of endometriosis patients in comparison with women without the disease [36]. Endometrial expression of interleukin-8 (IL-8) is responsible for the chemotaxis of neutrophils and partly for angiogenesis. The density of IL-8 receptors is significantly higher in women with endometriosis, as this molecule is involved in endometrial cell proliferation and attachment [17,23]. In a systematic review of different chemokines as markers of endometriosis, IL-8 appeared to be the most significant [9].

The anti-apoptotic *BCL-2* gene, upregulated in the eutopic endometrium of women with endometriosis, enhances cell survival and thus plays a major role in the pathogenesis of endometriosis. Increased proliferation and decreased apoptosis rates in the eutopic endometrium correlate with the expression profile of the *BCL-2* gene in endometriosis patients [37].

The endometriotic lesion cells express high levels of P450 aromatase–a protein which allows estrogen overproduction and decreases the expression of 17β-HSD2 (17β-Hydroxysteroid dehydrogenase), thus inhibiting the response to progesterone (“progesterone resistance”) [16]. This is considered a key process through which the maintenance and growth of endometriotic lesions are promoted. It is not known, however, whether these processes are a necessary cause of endometriosis or rather its consequence [32]. These results support the notion that intrinsic abnormalities in the eutopic endometrium cells in women with endometriosis predispose the endometriotic SCs cells to survive in the pelvic cavity, attach, invade, and establish a blood supply in the peritoneum or other areas.

Endometriotic lesions provoke local inflammation of the peritoneum, which attracts bmSCs through the expression of the C-X-C chemokine receptor type 4 (CXCR4) and of the chemokine ligand 12 (CXCL12) which plays a role of chemoattractant in the migration of bmSC towards the endometrial stromal cells. Thus, the deregulation of estrogen combined with local peritoneal injuries may be important in the pathogenesis of endometriosis [23]. Also, bmSCs may migrate from the peripheral circulation and provoke the formation of endometriosis foci in remote sites as well as infiltrate the endometrium of endometriotic lesions [19].

The endometriosis implant can also result from the outgrowths of the dormant SCs disseminated in the pelvic lining during embryogenesis of the female reproductive system [19] (see also Section 1).

Thus, pelvic and extrapelvic endometriosis implants are hypothesized, each with a distinctive epigenetic expression profile. Epigenetics plays a major role in modulating steroid action, and the inflammatory reaction is a key factor for the recruitment of bmSCs [5,38,39,40]. Whether gene expression profiles in endometriosis cells of the endometrium or bone marrow are similar or different remains unknown. Clarifying this puzzle is important to understand the pathogenetics of endometriosis.

## 3. Discussion

Genetic and epigenetic data analysis revealed significant differences in various tissues and cell types undergoing the EMDP compared to the normal ones. Complex molecular genetic and epigenetic features constitute the pathogenomic architecture of endometriosis and include gene polymorphisms, peculiarities of their expression, numerous interactions of gene nets, complex combinations of functional protein modules, as well as different metabolic pathways which are altered by sever imbalances in the hormonal and immunologic systems [3,5,32]. Each of these factors is affected at different levels during endometriosis depending on the specific EMDP. On the other hand, common clinical manifestations indicate the existence of some crucial molecular pathways common to all clinical types of endometriosis. Irrespective of the obvious differences in the intermediate events, the EMDP ultimately ends in the typical endometriotic lesions. Thus, the EMDP should be roughly subdivided into three parts: transition of mesodermal embryonic cells into cells of the endometrium within Muller ducts rudiments (1), acquisition of endometrial cells abnormalities and cell transition into endometriotic SCs (2), invasion of the SCs into the peritoneum lining and their differentiation into endometriotic lesions (3).

**Figure 1:**
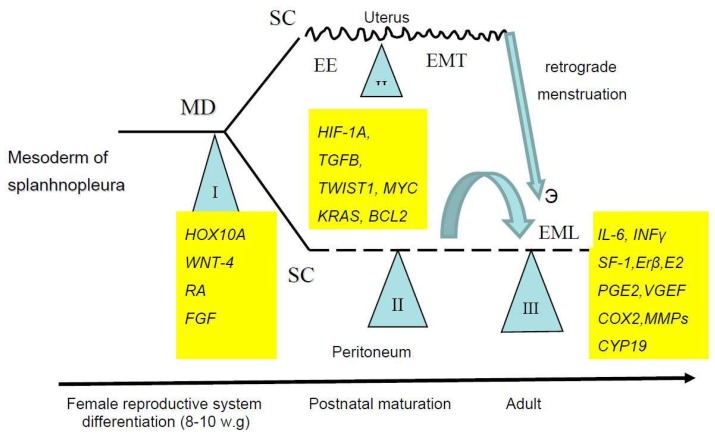
Sensitive periods in the Endometriosis Development Program. SC, stem cells, MD, Mullerian ducts, EE, eutopic endometrium, EMT, epithelial–mesenchymal transition, EML, endometriotic lesions, w.g., weeks of gestation.

As it was indicated (see 1), any developmental event should be attributed to a massive genome reprogramming which follows the short critical phases (the epigenetic crises after Waddington) of higher sensitivity to any inducers or noxious triggers [14,41]. Thus, at least three critical phases, corresponding to each of the morphogenetic events described above, should be recognized in the EMDP. The first one corresponds to the initial stages of the development of the reproductive tract in female embryos, while the second and third stages take place in postnatal life (Figure 1).

The dislocation of the primitive endometrial tissue in female fetuses coincides with human embryonic developmental stages XVII–XX (5–8 weeks of gestation) and lasts into the early postnatal period [42]. Both the coelomic epithelium of the peritoneum and the Mullerian ducts giving rise to all parts of the female reproductive tract generate from the mesoderm layer in the early human embryo. The development of the female urogenital tract is completed only at birth. The genes responsible for female reproductive tract development are well known, and many of them have already been identified [24]. The transcription factors of the *HOX* family, in particular *HOXA10*, are the principal coordinators and regulators of the expression of these genes [3], being responsible for mesoderm segmentation and its axial extension. The next important contributor to the formation of the Mullerian ducts is the *WNT* gene family, with *WNT4* as a key regulator of female sex development. It is located at the 1p36 chromosomal region, wich variants may contribute to endometriosis susceptibility through abnormal differentiation of the female reproductive tract [24]. *WNT4* was shown to be expressed in the normal peritoneum, suggesting that endometriosis can arise through a reversible transformation of the epithelium cells to endometriotic cells (metaplasia) through the developmental pathways associated with the *HOXA9* and *CDKN1A* genes [43]. These data are in line with a recently suggested “unifying hypothesis” of endometriosis [24]. According to this, Müllerian remnants of the endometrium may leak into the peritoneal cavity during embryogenesis of the urogenital system as a result of the deregulation of *WNT* genes and of the Wnt–β-catenin signaling pathway. The latter can lead to aberrations and deregulation within the mesoderm, thus causing the aberrant placement of SCs. Deregulation in the hormonal and immune systems, abnormalities of adhesion, extracellular matrix metalloproteinases, and pro-inflammatory cytokines activate or alter the peritoneal microenvironment, creating the conditions for the differentiation, adhesion, proliferation, and survival of ectopic endometrial cells, thus giving rise to endometriosis in adults. The growth of endometriotic lesions may occur by inclusion and transformation of the mesothelium cells of the peritoneal lining.

Structural variations (polymorphisms) or functional insufficiency of the *HOXA10* and *WNT4* genes and of the genes of their genetic cascade (*MIF*, *VEGFA*, *MMPs*, *VCAM*, *BMP*, etc.) may deregulate highly balanced genetic and epigenetic mechanisms of female reproductive tract embryogenesis, causing disorganization of the endometrium as well as dissemination of mesoderm cells, including SCs, outside the uterine cavity; this initiates an inborn predisposition to endometriosis in postnatal life. Mullerian embryogenesis-related genes in the uterine endometrium in early life might be associated with endometriosis in the adults.

Direct association of the *HOX* and *WNT* families as well as of 10 other genes with endometriosis was repeatedly confirmed [3,32]. By means of genome-wide association studies (GWAS), 12 single nucleotide polymorphisms at 10 independent genetic loci associated with endometriosis have also been identified [4]. Obviously, mesoderm cells with epigenetic or inborn defects incorporated both in the peritoneal lining and the uterine rudiments are suspected to be associated with the risk of developing endometriosis in adulthood [32].

Thus, endometriosis might be provoked by the failure of the expression of *HOXA10* or *WNTs* genes regulating the initial stages of reproductive tract development in female embryos or also induced by the direct harmful effects of some toxins during embryonic development, which result in the dislocation of the primitive endometrial tissue outside the uterine cavity during early organogenesis [44].

It also might be suspected that endometriotic SCs with inherited disorders of *WNT4* or *HOXA10* genes give rise to clinically forms of endometriosis more severe than those of mostly epigenetic origin [2].

Thus, the first sensitive period (SP) of the EMDP most probably corresponds to the embryonic stages of the female reproductive tract development. An unfavorable combination of endometriosis predisposition genes (predominantly of *WNT* and *HOX* families) and noxious agents (oxidative stress, pesticides, endocrine disruptors) might create conditions for the differentiation, adhesion, proliferation, and survival of eutopic and ectopic endometrial SCs. The direct association of the unfavorable *WNT4* allele with endometriosis has been recently demonstrated [45]. This finding deserves further studies to establish if this allele can be a predictive biomarker of endometriosis.

The second SP of the EMDP concerns the presence of dormant endometriotic cells in the endometrium. The duration of this period is unknown, as progenitors of endometriotic cells may stay dormant for many years until some provocative stimuli trigger their metaplasia into endometriotic SCs. Numerous genetic and epigenetic factors are involved. It was suspected and recently shown that eutopic endometrium cells in endometriosis patients contain aberrantly expressed genes and exhibit deregulated pathways that predispose them to implantation, invasion, and migration outside the uterus [34]. Dysfunctional expression of the genes related to the Mullerian embryogenesis (see SP1) as well as epigenetic immuno-endocrine deregulation of genes in endometrium (*IL11*, *LIF*, *TGF-*β, *FKBP4*, *COX2*, *PGs*, *FOXO1*, and *C/EBPβ*) might appear critical to the development of endometriotic lesions [3,32].

The involvement of external triggers, such as transient hypoxia, chronic inflammation, and mechanic transduction, is also suspected. Transient hypoxia and inflammation induce the *HIF-1A* gene and mechanic transduction upregulate the expression of the *TWIST1* gene. Thus, any measures reducing hypoxia and mechanical stretch of the uterus might be useful in endometriosis prevention. The search for other genes and epigenetic factors in eutopic endometrium cells predisposing to endometriosis should be encouraged.

The third SP of the EMDP includes adhesion, proliferation, invasion, angiogenesis, and growth of endometriotic stem cells into endometriotic lesions. The genes highly expressed at this stage include cell cycle regulators (cyclins and CDKs), angiogenesis factors (*VEGFA*, *ANGPTs*, and *TIEs*), immuno-inflammatory factors (*COX2*), matrix metalloproteinases (*MMP3, MMP9*), and integrins. Their protein products play a critical role in the establishment, maintenance, and development of the endometriotic lesions. Theoretically, interference with the expression of any of this gene might be sufficient for the active prevention and treatment of endometriosis. Clinical practice, however, contradicts these assumptions and favors the view that the EMDP is a well-canalized process, buffered against curative intrusions. At a definite stage of progression, the EMDP becomes irreversible and proceeds to its final stage producing the endometriotic lesions. It should be mentioned that in women receiving a hormonal contraceptive treatment that prevents the implantation, the frequency of endometriotic lesions on the peritoneum is comparable with that of the controls [46]. In agreement with this, hormonal treatment did not prevent the invasion and implantation of endometriotic SCs. On the other hand, to the best of our knowledge, the implantation of endometriotic SCs per se as well as their invasion into the pelvic lining was never registered, thus giving some credit to the extra uterine origin of endometriosis from the mesenchymal stem cells (meSC) disseminated during embryogenesis of the female reproductive tract (See part 1).

## 4. Conclusions

As might be inferred from the reviewed studies and suggested hypothesis, each of the three sensitive stages in the EMDP deserves special attention. Intrinsic and external factors interfering with the embryogenesis of the female reproductive tract should be subjected to thorough studies. Of special interest are the inherited forms of endometriosis and their correlation with relevant mutations or polymorphisms of the genes involved in the differentiation of the Mullerian duct and in the development of the urogenital tract, such as *WNT*, *HOXA10*, *HOXA11*, and their signaling pathways, as well as other genes regulating mesoderm differentiation and SC trafficking. The search for teratogenic agents affecting the development of the female reproductive tract should also be encouraged.

More knowledge of SP2 should be drawn from the data on the heterogeneity of eutopic endometrium cells, with special emphasis on the cells prone to induce endometriotic lesions growth. The significance of EMT as a trigger of epigenetic changes amenable to launch the EMDP should be also considered. Both SP1 and SP3 need further global molecular studies of gene expression and its regulation by methylation and microRNA analysis. There are still few reports on these topics, with rather contradictory results for both endometrial transcriptome [9,47] and microRNAs [10]. Large differences between studies can be explained by differences in the study design, subject characteristics, procedures for tissue collection, storage, and processing, assay platforms and data analysis methods. The necessity for the unification of these variables was recently supported by the World Endometriosis Research Foundation initiative that issued the Endometriosis Phenome and Biobanking Harmonization Project, which developed standards for tissue collection, processing, and storage in endometriosis research [48]. It looks very awarding that only –omics analysis of massive endometriosis data stratified according system genetics architecture and collected according to International Conference on Bioinformatics and Biomedicine regulations [7] may pave a reliable way to ultimate solution of endometriosis mystery and maybe give more credit to existence of special developmental program in pathogenomics of endometriosis.

## Abbreviations

EMDP	Endometriosis development program
ESC	Endometrial stem cells
SC	Stem cells
SP	Sensitive period
bmSC	Bone marrow stem cells
meSC	Mesenchymal stem cells
EMT	Epithelial–mesenchymal transition

## References

[1] Batt R.E. (2011). A History of Endometriosis.

[2] Shubina A.N., Egorova A.A., Baranov V.S., Kiselev A.V. (2013). Recent advances in gene therapy of endometriosis. Recent Pat. DNA Gene Seq..

[3] Borghese B., Zondervan K.T., Abrao M.S., Chapron C., Vaiman D. (2017). Recent insights on the genetics and epigenetics of endometriosis. Clin. Genet..

[4] Zondervan K.T., Rahmioglu N., Morris A.P., Nyholt D.R., Montgomery G.W., Becker C.M., Missmer S.A. (2016). Beyond endometriosis GWAS: From Genomics to Phenomics to the Patient Europe PMC Funders Group. Semin. Reprod. Med..

[5] Grimstad F.W., Decherney A. (2017). A Review of the Epigenetic Contributions to Endometriosis. Clin. Obstet. Gynecol..

[6] Liu J., Zhao M. (2016). A PubMed-wide study of endometriosis. Genomics.

[7] Akter S., Wilshire G., Davis J.W., Bromfield J., Crowder S., Pelch K., Meng A., Barrier B., Nagel S.C. A Multi-Omics Informatics Approach for Identifying Molecular Mechanisms and Biomarkers in Clinical Patients with Endometriosis. Proceedings of the IEEE International Conference on Bioinformatics and Biomedicine (BIBM).

[8] Daraï E., Ploteau S., Ballester M., Bendifallah S. (2017). Endométriose: Physiopathologie, facteurs génétiques etdiagnostic clinique. La Presse Médicale.

[9] Aghajanova L., Burney R.O., Tran N.D., Giudice L.C. (2017). mRNA and miRNA Biomarkers for Endometriosis In Biomarkers for Endometriosis.

[10] Saare M., Rekker K., Laisk-podar T., Rahmioglu N., Salumets A., Martin G., Peters M. (2017). Challenges in Endometriosis MiRNA Studies—From Tissue Heterogeneity to Disease Specific MiRNAs. Biochim. Biophys. Acta.

[11] Krishnamoorthy K., Decherney A.H. (2017). Genetics of Endometriosis. Clin. Obstet. Gynecol..

[12] Sapkota Y., Steinthorsdottir V., Morris A.P., Fassbender A., Rahmioglu N., De Vivo I., Buring J.E., Zhang F., Edwards T.L., Jones S. (2017). Meta-analysis identifies five novel loci associated with endometriosis highlighting key genes involved in hormone metabolism. Nat. Commun..

[13] Stockard C.R. (1921). Developmental rate and structural expression: An experimental study of twins ‘double monsters’ and single deformities, and the interaction among embryonic organs during their origin and development. Dev. Dyn..

[14] Saxen L., Rapila J., Ebert J.D. (1969). Sensitive periods in development. Congenital Defects.

[15] Baranov V.S., Ivaschenko T.E., Liehr T., Yarmolinskaya M.I. (2014). Systems genetics view of endometriosis: A common complex disorder. Eur. J. Obstet. Gynecol..

[16] Gargett C.E., Gurung S. (2016). Endometrial Mesenchymal Stem/Stromal Cells, Their Fibroblast Progeny in Endometriosis, and More. Biol. Reprod..

[17] Santamaria X., Massasa E.E., Taylor H.S. (2012). Migration of cells from experimental endometriosis to the uterine endometrium. Endocrinology.

[18] Proestling K., Birner P., Balendran S., Nirtl N., Marton E., Yerlikaya G., Kuessel L., Reischer T., Wenzl R., Streubel B. (2016). Enhanced expression of the stemness-related factors OCT4, OX15 and TWIST1 in ectopic endometrium of endometriosis patients. Reprod. Biol. Endocrinol..

[19] Lagana A.S., Salmeri F.M., Vitale S.G., Triolo O., Gotte M. (2017). Stem Cell Trafficking During Endometriosis: May Epigenetics Play a Pivotal Role?. Reprod. Sci..

[20] Valentijn A.J., Palial K., Al-Lamee H., Tempest N., Drury J., Von Zglinicki T., Saretzki G., Murray P., Gargett C.E., Hapangama D.K. (2013). SSEA-1 isolates human endometrial basal glandular epithelial cells: Phenotypic and functional characterization and implications in the pathogenesis of endometriosis. Hum. Reprod..

[21] Meng X., Ichim T.E., Zhong J., Rogers A., Yin Z., Jackson J., Wang H., Ge W., Bogin V., Chan K.W. (2007). Endometrial regenerative cells: A novel stem cell population. J. Transl. Med..

[22] Baranov V.S., Ivaschenko T.E., Yarmolinskaya M.I. (2016). Comparative systems genetics view of endometriosis and uterine leiomyoma: Two sides of the same coin?. Syst. Biol. Reprod. Med..

[23] Wang X., Mamillapalli R., Mutlu L., Du H., Taylor H.S. (2015). Chemoattraction of bone marrow-derived stem cells towards human endometrial stromal cells is mediated by estradiol regulated CXCL12 and CXCR4 expression. Stem Cell Res..

[24] Laganà A.S., Vitale S.G., Salmeri F.M., Triolo O., Ban Frangež H., Vrtačnik-Bokal E., Stojanovska L., Apostolopoulos V., Granese R., Sofo V. (2017). Unus pro omnibus, omnes pro uno: A novel, evidence-based, unifying theory for the pathogenesis of endometriosis. Med. Hypotheses.

[25] Sampson J.A. (1927). Peritoneal endometriosis due to menstrual dissemination of endometrial tissue into peritoneal cavity. Am. J. Obstet. Gynaecol..

[26] Yang Y.M., Yang W.X. (2017). Epithelial-to-mesenchymal transition in the development of endometriosis. Oncotarget.

[27] Kalluri R., Weinberg R.A. (2009). The basics of epithelial-mesenchymal transition. J. Clin. Investig..

[28] Lamouille S., Xu J., Derynck R. (2014). Molecular mechanisms of epithelial-mesenchymal transition. Nat. Rev. Mol. Cell Biol..

[29] Young V.J., Brown J.K., Saunders P.T., Duncan W.C., Horne A.W. (2014). The peritoneum is both a source and target of TGF-β in women with endometriosis. PLoS ONE.

[30] Tseng J.C., Chen H.F., Wu K.J. (2015). A twist tale of cancer metastasis and tumor angiogenesis. Histol. Histopathol..

[31] Brunet T., Bouclet A., Ahmadi P., Mitrossilis D., Driquez B., Brunet A.C., Henry L., Serman F., Béalle G., Ménager C. (2013). Evolutionary conservation of early mesoderm specification by mechanotransduction in Bilateria. Nat. Commun..

[32] Ito F., Yamada Y., Shigemitsu A., Akinishi M., Kaniwa H., Miyake R., Yamanaka S., Kobayashi H. (2017). Role of oxidative stress and epigenetic modification in endometriosis. Reprod. Sci..

[33] Capobianco A., Rovere-Querini P. (2013). Endometriosis, a disease of the macrophage. Front. Immunol..

[34] Kobayashi H., Iwai K., Niiro E., Morioka S., Yamada Y. (2014). Fetal programming theory: Implication for the understanding of endometriosis. Hum. Immunol..

[35] Koippallil Gopalakrishnan Nair A.R., Pandit H., Warty N., Madan T. (2015). Endometriotic mesenchymal stem cells exhibit a distinct immune phenotype. Int. Immunol..

[36] Young V.J., Ahmad S.F., Brown J.K., Duncan W.C., Horne A.W. (2015). Peritoneal VEGF-A expression is regulated by TGF-β1 through an ID1 pathway in women withendometriosis. Sci. Rep..

[37] Barragan F., Irwin J.C., Balayan S., Erikson D.W., Chen J.C., Houshdaran S., Piltonen T.T., Spitzer T.L., George A., Rabban J.T. (2016). Human Endometrial Fibroblasts Derived from Mesenchymal Progenitors Inherit Progesterone Resistance and Acquire an Inflammatory Phenotype in the Endometrial Niche in Endometriosis. Biol. Reprod..

[38] Pluchino N., Taylor H.S. (2016). Endometriosis and Stem Cell Trafficking. Reprod. Sci..

[39] Koukoura O., Sifakis S., Spandidos D.A. (2016). DNA methylation in endometriosis. Mol. Med. Rep..

[40] Bulun S.E., Monsivais D., Kakinuma T., Furukava Y., Barnardi L., Pavone M.E., Dyson M. (2015). Molecular biology of endometriosis^ from aromatase to genomic abnormalities. Semin. Reprod. Med..

[41] Waddington C.H. (1968). Tendency towards regularity of development and their genetical control. International Workshop Teratology.

[42] Carlson B.M. (2009). Human Embryology and Developmental Biology.

[43] Gaetje R., Holtrich U., Engels K., Kissler S., Rody A., Karn T., Kaufmann M. (2007). Endometriosis may be generated by mimicking the ontogenetic development of the female genital tract. Fertil. Steril..

[44] Signorile P.G., Baldi F., Bussani R., Viceconte R., Bulzomi P., D’Armiento M., D’Avino A., Baldi A. (2012). Embryonic origin of endometriosis: Analysis of 101 human fetuses. J. Cell. Physiol..

[45] Matalliotakis M., Zervou M.I., Matalliotaki C., Rahmioglu N., Koumantakis G., Kalogiannidis I., Prapas I., Zondervan K., Spandidos D.A., Matalliotakis I. (2017). The role of gene polymorphisms in endometriosis. Mol. Med. Rep..

[46] McKinnon B.D., Bertschi D., Wanner J., Bersinger N.A., Mueller M.D. (2014). Hormonal Contraceptive Use and the Prevalence of Endometriotic Lesions at Different Regions within the Peritoneal Cavity. Biomed. Res. Int..

[47] Zhao L., Gu C., Ye M., Zhang Z., Han W., Fan W., Meng Y. (2017). Identification of global transcriptome abnormalities and potential biomarkers in eutopic endometria of women with endometriosis: A preliminary study. Biomed. Rep..

[48] Fassbender A., Rahimoglu N., Vitonis A.F., Viganò P., Giudice L.C., D’Hooghe T.M., Hummelshoj L., Adamson G.D., Becker C.M., Missmer S.A. (2014). World Endometriosis Research Foundation Endometriosis Phenome and Biobanking Harmonisation Project: IV. Tissue collection, processing, and storage in endometriosis research. Fertil. Steril..

